# Occurrence of protein structure elements in conserved sequence regions

**DOI:** 10.1186/1472-6807-7-3

**Published:** 2007-01-09

**Authors:** Einat Sitbon, Shmuel Pietrokovski

**Affiliations:** 1 Department of Molecular Genetics, Weizmann Institute of Science, Rehovot, Israel

## Abstract

**Background:**

Conserved protein sequence regions are extremely useful for identifying and studying functionally and structurally important regions. By means of an integrated analysis of large-scale protein structure and sequence data, structural features of conserved protein sequence regions were identified.

**Results:**

Helices and turns were found to be underrepresented in conserved regions, while strands were found to be overrepresented. Similar numbers of loops were found in conserved and random regions.

**Conclusion:**

These results can be understood in light of the structural constraints on different secondary structure elements, and their role in protein structural stabilization and topology. Strands can tolerate fewer sequence changes and nonetheless keep their specific shape and function. They thus tend to be more conserved than helices, which can keep their shape and function with more changes. Loop behavior can be explained by the presence of both constrained and freely changing loops in proteins. Our detailed statistical analysis of diverse proteins links protein evolution to the biophysics of protein thermodynamic stability and folding. The basic structural features of conserved sequence regions are also important determinants of protein structure motifs and their function.

## Background

What are the structurally distinguishing features of conserved protein sequence regions? The structure of many diverse proteins is currently known. In addition, many more protein sequences have been determined. These data can be used to study the relations between structure and conserved sequence features of proteins, such as protein secondary structure, which is a basic structural attribute that defines structural folds.

Sequence conservation of homologous sequences is rarely homogenous along their length; as sequences diverge, their conservation is localized to specific regions. Typically, evolutionary conserved regions are important both structurally and functionally. To obtain the general structural features of conserved regions of all proteins, it is necessary to decide which scale of protein clustering, conserved regions, and structure features to analyze. Natural choices are generically defined protein families [1], ungapped protein sequence motifs (blocks) that separate proteins into either conserved or random regions [2], and the four basic secondary structure elements (SSEs), namely, alpha helices, beta strands, structured turns, and loops [3]. It is also of paramount importance to analyze data from a very large and diverse group of proteins, avoiding conclusions drawn from biased and a limited amount of data and using exact statistics to identify subtle but significant features.

Using blocks as the basic unit of analysis is advantageous over other precomputed multiple sequence alignments since the modular nature of proteins naturally lends itself to description by motifs; that is locally conserved sequence regions that include at least a few invariant positions [4]. While specific families are typically described by groups of motifs, each motif is not necessarily associated with only one single family. A motif can appear in different contexts in separate families and be repeated within one family (for an example see [5]). The conservation of the known family members defines the number and length of its motifs [1].

Relations between protein sequence and structure can be analyzed by either determining the sequence features of predefined structures, as reviewed by Bystroff [6], or by determining structural features of conserved sequence regions. Han and Baker studied local structure features that predominate short sequence motifs, identifying correlations between specific sequence and structure motifs [7]. Secondary structure conservation was previously studied in structural alignments of protein families and SSE substitution matrices were created [8]. The conservation of SSEs was also studied in some specific protein families (e.g. [9]). Protein loops and their flanking regions were found to be conserved to the same extent in an analysis of a large set of proteins [10].

Protein structures can be divided into four major structural classes, according to their secondary structure content and arrangement (SCOP [11]). There are two homogeneous classes and two heterogeneous classes. The homogenous classes consists of structures containing mainly alpha helices (termed all alpha) or containing mainly beta strands (all beta). The two heterogeneous classes comprise both alpha helices and beta strands. The alpha/beta class consists of mainly parallel beta sheets (beta-alpha-beta units), and the alpha+beta class that consists of mainly antiparallel beta sheets (segregated alpha and beta regions) [11]. Each class obviously differs in its secondary structure content. An analysis of SSE occurrences should therefore control for the possible bias created by the different representations of each class.

Proteins with similar sequences adopt similar structure [12,13]. However, similar structures can have less than 12% sequence identity [11,14-16]. Most amino acids within a protein can thus be changed without affecting its structure, including the secondary structure [17]. Previous experiments have shown that both helices and strands can undergo numerous mutations and still keep their secondary structure – either beta-strand or alpha helix – and also maintain structural stability [18-20]. The number of point mutation per position that can be tolerated by a protein without loosing structural stability can be represented by a neutral network. Protein neutral network models have shown that stable structures have a large neutral network [21-23]. Neutral networks may also be analyzed empirically, using conserved regions and regions from random locations on proteins. Such an analysis would emphasize the connection between conservation in actual protein families and the stability of protein structures.

Exposure to the solvent is known to be anti-correlated with conservation; that is, core residues, particularly polar core residues, are usually highly conserved [24,25]. Therefore, differences in conservation of SSEs might be due to differences in accessibility to the solvent. Since conservation, solvent accessibility and secondary structures are interrelated, an analysis of conservation should consider the possible effect of surface accessibility.

Helices and strands have a regular repetitive structure, while turns and loops are not repetitive [26]. This suggests that helices and strands might be more conserved than loops and turns. On the other hand, functional sites were shown to be overrepresented in loop-rich regions [10], suggesting that loops may tend to be evolutionarily conserved.

The findings of our analysis were somewhat surprising and have not been noted in other studies. Helices and turns were found to be underrepresented in conserved regions, unlike strands, which were found to be overrepresented. Loops were found in similar numbers in both conserved and random regions. The significant differences in the occurrence of SSEs between conserved regions and random regions was found in a large set of protein sequences. This can facilitate a clearer understanding of the relationship between evolution and structure. Our detailed analysis of SSEs, in all protein structure types as well as in separate structural classes, shows how the combination of sequence and structural features indicates functionally and structurally important regions. This is an advantage when designing new proteins, as well as for studying structures that do not have any known homologs.

## Results

### Conserved regions include more beta strands than expected and fewer alpha helices and turns

The relation between structure and conserved sequence features was examined by establishing the secondary structure element (SSE) distribution in either conserved or random sequence regions. Four SSEs were analyzed – helix, strand, loop and turn (including hydrogen bonded turn) based on the DSSP definitions [3]. The conserved regions used were blocks From the Blocks database [2,27], that is, locally aligned, highly conserved ungapped protein regions. SSEs were analyzed for a large number of protein families, each containing one or several blocks, and at least one known protein structure (Table 1). Information on sequence and structure was analyzed for all protein families in order to identify subtle features in a statistically significant manner.

**Figure 1:**
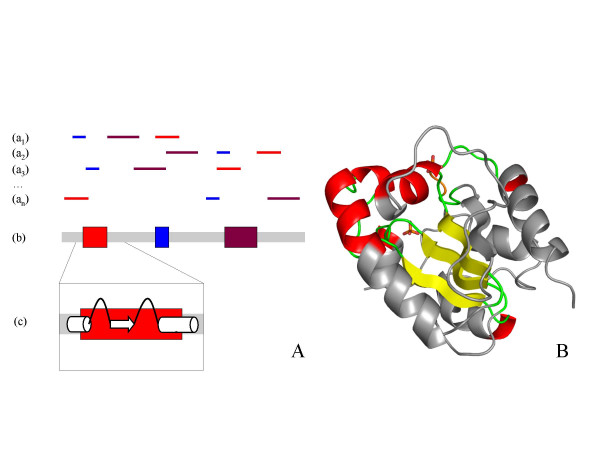
**A scheme of the analysis approach and a sample structure.** (A) Counting secondary structure elements in conserved protein regions and estimating their occurrence in all of the protein. A sample protein is shown with three conserved regions in color (b), and the secondary structure assignment for one of the regions (c). Sampling of random regions with the same widths as the conserved regions is schematically shown in (a_1-n_). (B) Structure of MUG G:T/U mismatch-specific DNA glycosylase (PDB Id 1mug) in cartoon representation, with conserved regions colored by secondary structure (strand yellow, loop green, helix red, and turn orange). There are relatively more strands in conserved regions than helices: three out of four strands are in conserved regions, while four out of seven helices are in conserved regions.

Background occurrences of SSEs were found from sets of same-size regions in random locations on the same proteins as the set of conserved regions (Figure 1(A)). The unit counted was the appearance of each SSE, regardless of its length. This unit was chosen in order to avoid inconsistencies in defining the ends of SSEs, and to decrease the effect of the different lengths of the analyzed conserved regions. In Addition, some SSEs can be by definition longer or shorter, for instance helices are at least three residues long, while loops are assigned to very long stretches that have no defined secondary structure. The distribution of the SSEs in the conserved regions was found to differ significantly from that in random regions (A chi square goodness-of-fit test of X^2^_(3) _= 91.2 and p-value 1.2E-19, Table 2).

**Figure 2:**
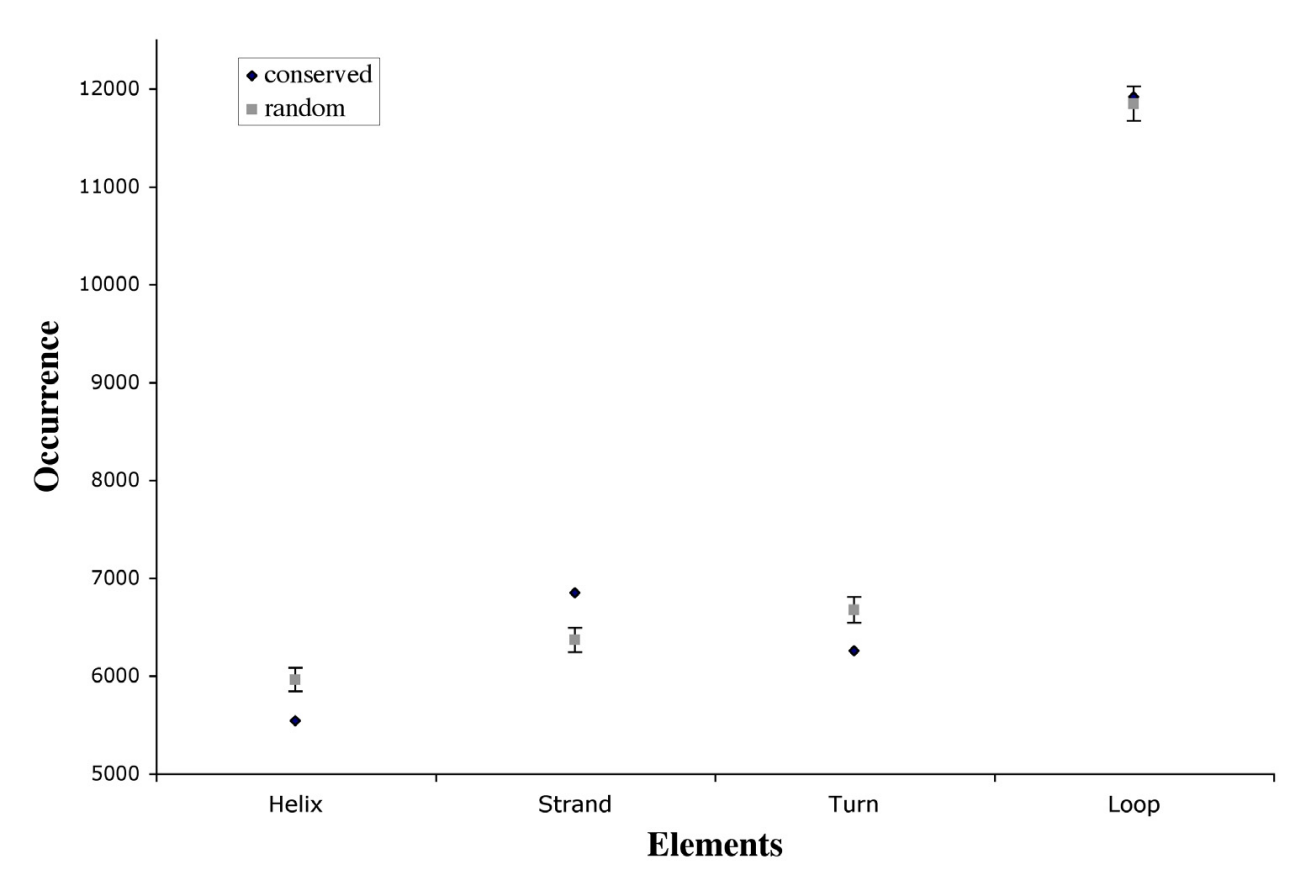
**Occurrence of secondary structure elements (SSEs) in conserved vs. random regions. **The random value is the average of twenty-two sets, with a prediction interval. Prediction intervals were created by the standard deviation of the random sets multiplied by the t value for 21 degrees of freedom for a confidence of at least 95%.

Next, each SSE type was tested separately in order to analyze its contribution to the observed difference SSEs between conserved and random regions. Our null hypothesis was that the occurrence of each SSE in the conserved regions belongs to the distribution of the same SSE in random regions (represented by several such sets). Helix, strand and turn SSEs in conserved regions were found outside the corresponding prediction interval for 95% significance, as seen in Figure 2. The actual differences are shown in Table 3 and confirm the cell chi square values shown in the analysis in Table 2. The differences are not very large – between 6.7% and 7.5% – but they are significant.

Hence, in comparing the presence of SSEs between conserved regions and all the protein (represented by random region units), strands are significantly more common in conserved regions, while helices and turns are less common in conserved regions, and loops appear similarly in both types of regions (see example in Figure 1(B)).

### SSE conservation in separate structural classes and folds

**Figure 3:**
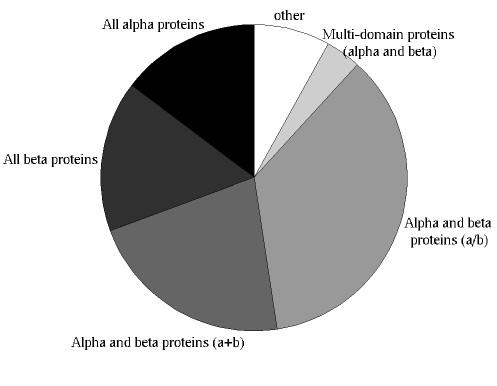
**Distribution of conserved regions among protein classes**. Fraction of different conserved regions (blocks) in each protein class relative to all analyzed blocks. The "other" category includes classes with a small number of examples, such as coiled coils or membrane and cell surface proteins and peptides, as well as blocks that belong to two adjacent domains.

**Figure 4:**
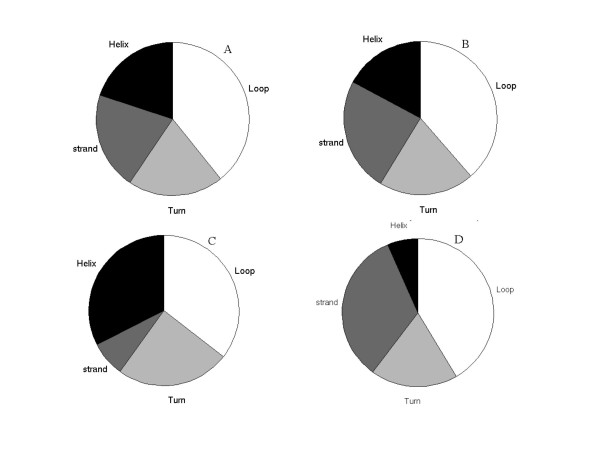
**Secondary structure distribution in conserved regions of the different protein classes**. (A) Alpha/Beta proteins, (B) Alpha+Beta proteins, (C) all Alpha proteins, (D) all Beta proteins.

To ensure our results are true, we controlled for structural class, solvent accessibility and protein size. In order to determine if the observed over – and under – abundance of some SSEs was due to an overrepresentation in one structural class, the dataset was divided into the four major protein classes: alpha+beta, alpha/beta, all alpha and all beta proteins (SCOP [11]). Most of the analyzed regions belong to one of the heterogeneous classes – alpha+beta and alpha/beta (Figure 3). In each class, SSEs are distributed differently (Figure 4). It should be noted that the all alpha and all beta classes contain some beta and alpha SSEs respectively, in contrast to what their names imply. Next, the occurrences of helices and strands in conserved vs. random regions were examined for each structural class separately to determine if the differences found for all protein types together persist in each class instance.

**Figure 5:**
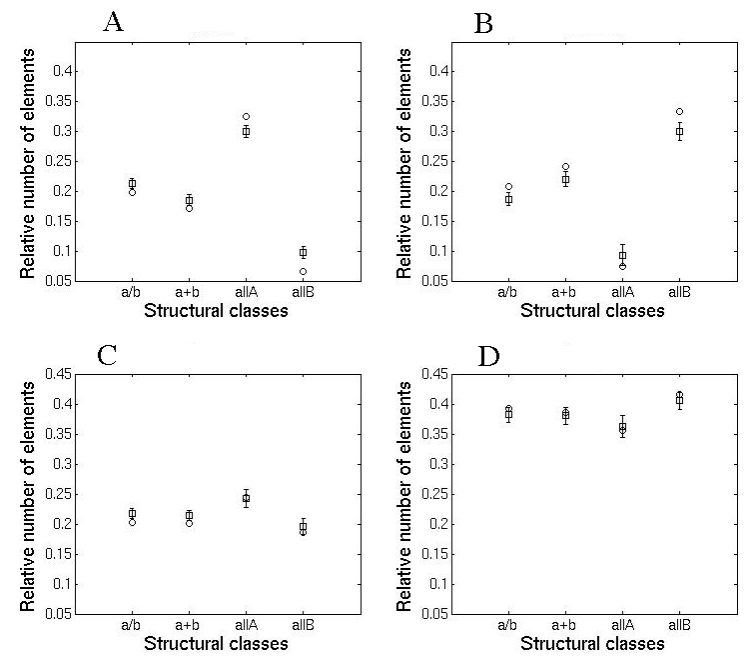
**Relative occurrence of each secondary structure element (SSE) in conserved vs. random regions**. Conserved regions marked as a cross, and random regions marked as a square with a prediction interval. The random regions value is the average of twenty-two sets, with a confidence interval created by the standard deviation of the random sets multiplied by the t value for 21 degrees of freedom. (A) Helices; (B) Strands; (C) Turns; (D) Loops.

The occurrences of helix, strand, loop and turn in conserved regions were found to be significantly dependent on their structural class (Figure 5, Table 4). Helices were significantly less frequent in conserved regions in three of the four structural classes. The all-alpha class was the exception, since helices were significantly (although by a small margin) more frequent in conserved regions. The all-alpha class also differed from the other classes for strands. This is the only class where strands were less common in conserved regions. Each of the four classes had different results for turns and loops (Figure 5). From this analysis, we conclude that the overrepresentation of strands and underrepresentation of helices in conserved regions, is weakly related to structural class. The SSE deviations found were thus probably an outcome of a universal, rather than class-specific process.

### Solvent accessibility, conservation and SSEs

To check if differences in conservation of SSEs were due to differences in accessibility to the solvent, the relation between accessibility and SSE distribution was examined for conserved regions in two ways. First, the properties of whole conserved regions were analyzed. In addition, the accessibility of each residue in conserved regions was analyzed and compared to residues from whole proteins.

**Figure 6:**
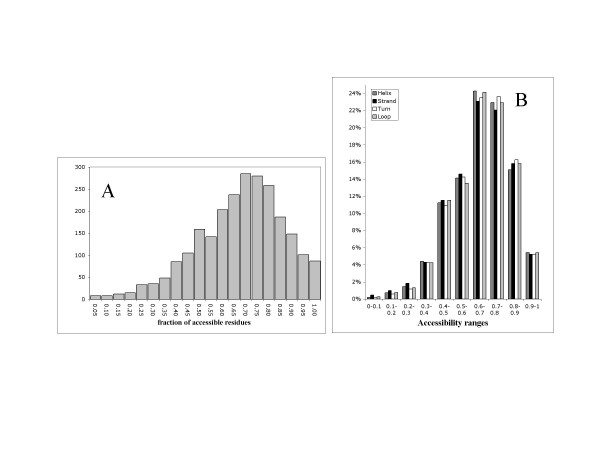
**Block accessibility distribution**. **A.** Accessibility distribution among   all analyzed blocks. **B**. Relative frequency of each SSE in accessibility  ranges (for example, there are less than one percent of the strands in the 0–0.1 block accessibility range).

The distribution of SSE frequencies over several accessibility ranges was examined. The accessibility of a region was defined by the fraction of the solvent exposed (>5% accessibility) amino acids in that region. It should be noted that although each residue is taken to be either accessible or buried in this analysis, the accessibility of the region is typically a fraction. For example a conserved region that is 33% accessible has about a third of its residues at the surface of the protein, while the remaining two thirds are not accessible to the solvent. Ninety percent of all the analyzed regions had an average accessibility of more than forty percent (Figure 6A). The four SSEs were found to distribute evenly over different accessibility ranges (Chi^2^_(24) _= 17.6 P value = 0.8 for independence of SSE frequencies and solvent accessibility: Figure 6B). When each of the four structural classes were examined separately (not shown), the SSEs were found to also distribute evenly over accessibility ranges. This analysis of the correlation between secondary structure and accessibility indicates that differences in conservation of SSEs are not only due to differences in accessibility.

In order to verify that the small difference in helix versus strand distributions in the low accessibility ranges does not affect the significance of the results, the extreme case in which buried strands occur only in conserved regions was assumed. Therefore, only the number of accessible strands in conserved regions was compared to the number of strands in random regions. There are 6846 strands in conserved regions and 6362 strands in random regions. This difference of 484 strands corresponds to 3.51 confidence intervals (Tables 2 and 3). Not counting the 126 buried strands of conserved regions (with an average accessibility of 30%) reduces the difference to 358 strands, which corresponds to 2.59 confidence intervals. The difference between the number of strands in conserved and random regions is therefore significant even when all the buried conserved regions strands are not considered. In other words, the significant difference in the number of strands between conserved and random regions is not only due to buried conserved regions.

A second analysis checked whether strand residues remain more common in conserved regions even when they are exposed to the solvent, regardless of the average accessibility of the whole region. Unlike the previous analysis, here information on which elements within a conserved region were exposed, and which were buried was retained. The odds that an accessible residue would be a strand in conserved regions and in the whole protein were calculated. The log ratio of these two odd values was calculated as 0.15 ± 0.02, which is slightly, but significantly above zero. This means that strands are slightly, although significantly more common in accessible conserved residues relative to accessible residues in the whole protein.

To further validate our findings, block-sized regions were sampled in random locations in proteins, and their amount of accessible strands compared to that in block regions. Normalizing the number of accessible residues in conserved regions to the total number of accessible residues, 9226.2 accessible strand residues were found in the examined conserved regions and 7983.1 accessible strand residues were found in an average of twenty sets of random regions from the same proteins. The confidence interval for the average random regions sets is 468.5. Thus, conserved regions were found to have significantly more accessible strand regions – by 2.65 more confidence intervals. This analysis confirms that accessible strands are more common in conserved regions than in the whole protein.

The above analyses establish that the higher abundance of strands in conserved regions is independent from accessibility.

**Figure 7:**
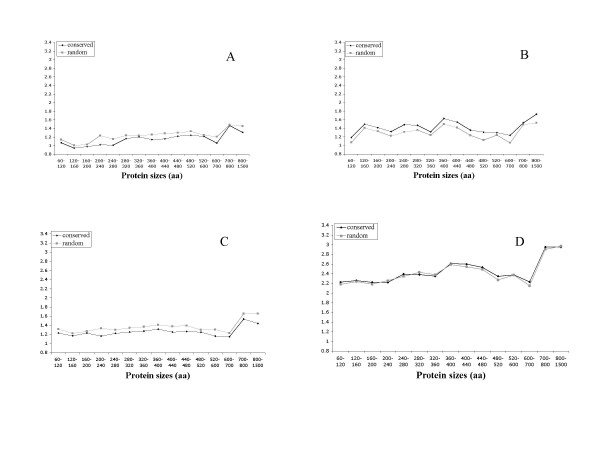
**Relative frequency of number of SSE types in different size bins**. The average number of each SSE per block in each size range. The number of blocks per range varies between 275 and 601. (A) Helices in conserved and random regions; (B) Strands; (C) Turns; (D) Loops.

In order to test the robustness of our results, conserved regions were to divided into bins according to the size of the proteins in which they occur (Figure 7). There are consistently more strands and fewer helices and turns in conserved regions than in random regions in proteins of all sizes, although the differences are not always significant. No consistent differences were found in loop occurrences. Abundance of secondary structure in conserved regions therefore does not depend on protein size. Dividing the data into bins according to protein size also shows the general robustness of our findings.

The small difference in strand, helix and turn occurrences is significant when all proteins in the database were analyzed. This was also true when the proteins were separated into groups according to size and when accessibility was considered. Except for the all-alpha protein class, the same results were obtained for separate protein classes. This shows a real effect in the preference of secondary structure in conserved regions.

## Discussion

In this study we present a novel approach that links evolutionary conservation to protein secondary structure. This large scale analysis allowed us to draw general conclusions on the relation between biophysical aspects of protein folding and sequence space.

We have identified significant differences in SSE occurrence between conserved and random sequence regions. Strands are overrepresented in conserved regions, while helices and turns are underrepresented. While these are significant differences, they are also not large – around 7% difference for all proteins (Table 3), and up to a 10% difference for individual protein classes (Table 4).

In defining conserved regions we focused on highly conserved blocks. Conservation is dependent on the size and variability of a protein family. Therefore, a measure of conservation, consistent across different families is not available. We chose the all-or-none definition of conservation of the Blocks database, knowingly leaving out weakly conserved regions. Our definition created a set of highly conserved sequence regions and a set of sequence regions with the average protein conservation. One consequence of the unavoidable mixture of conserved and non-conserved protein regions is identification of trends, rather than exact values, for conserved region features. Therefore, the differences between conserved and non-conserved regions might actually be stronger than what we observed when analyzing the most conserved regions.

The measured unit in this study was a SSE, disregarding its length. This unit enabled us to compare SSEs as they appear in the protein topology, and ignore phenomena that affect their length, such as the ambiguous structural nature of SSE ends, and insertions/deletions, that do not affect the topology of the protein.

Differences between conserved and random regions could be the result of heterogeneity in the data, due to factors such as structural classes, accessibility, active site regions and regions important for structural stabilization. Dividing the analyzed data to subsets according to structural classes, accessibility values, and protein sizes, while using various statistical methods, enhanced some of the observed differences, as specified below.

Several ways of neutralizing the effect of solvent accessibility did not change our results significantly. The tendency of strands to be more buried than helices is therefore not the main reason for the overabundance of strands in conserved regions.

Differences in SSE content were found between the four major structural classes, as well as between conserved and random regions of each class. We found a significant difference in the SSE content of the four major structural classes, and each class showed differences in SSE content between its conserved and random regions. Structural classes are defined by the content and order of their SSEs (e.g. all alpha proteins are mainly composed of alpha helices while alpha/beta proteins mainly consist of parallel beta sheets). However, the differences we found between the classes are neither trivial nor expected. First, the alpha/beta class is combined with the alpha+beta class in the CATH structural classification database [28], although we found these two classes to be clearly distinct, therefore agreeing with the class definitions suggested by Levitt and used in SCOP [29,11]. Our results show that the division of protein structures by topology and not SSE content alone is more meaningful. Next, in all classes, except the all-Alpha, strands and helices occurred in the same way. This general feature of strands and helices justifies drawing general conclusions about the constraints on their occurrences.

### A thermodynamics explanation of strands and helices occurrences in conserved regions

The nature of a protein depends on more than its secondary structure. Specific features of SSEs are necessary to create a specific protein trait. For example, SSEs in a specific active site require unique features, such as flexibility, distinct shape, and charge distribution. SSEs that could adopt these features with a limited number of sequence combinations tend to be conserved.

Two attributes that characterize helices and strands could explain our findings regarding the overrepresentation of strands and the underrepresentation of helices in conserved regions. The amino acid diversity in strands and helices is approximately the same [30,31]. However, in general, helices have a specific backbone conformation while strands are more diverse in shape. A specific helix can thus be created by many sequence combinations, while a specific strand structure requires a more limited set of sequence combinations. The Ramachandran plot shows that the space of possible backbone conformations of helices is more limited than that of strands [32]. In Addition, the bulky helix backbone reduces available degrees of freedom in helix side chains [33], and residues in alpha helix conformation were shown to have consistently lower entropies than residues in strand conformations [34]. Therefore, there is an entropic constraint – a reduction of degrees of freedom – for helix protein regions. This rigidity allows a larger variety of sequences to maintain a specific helical structure. On the other hand, strand flexibility requires additional sequence constraints in order to maintain a specific strand structure. An alpha helix region can therefore retain its specific tertiary structure with a higher variety of amino acid types than strands.

The above reasoning explains why strands are more likely to be conserved, and helices are less likely to be conserved. Our analysis examined what seems to be a similar question but is in fact not identical, namely, how likely conserved regions are to include strands or helices. Therefore, our findings regarding SSE occurrences in conserved regions could be explained by the conservation likelihood of SSEs.

That an alpha helix is less likely to be conserved can be formally defined by:

p(c|a)<p(c)⇒p(c|a)p(c)<1
 MathType@MTEF@5@5@+=feaafiart1ev1aaatCvAUfKttLearuWrP9MDH5MBPbIqV92AaeXatLxBI9gBaebbnrfifHhDYfgasaacH8akY=wiFfYdH8Gipec8Eeeu0xXdbba9frFj0=OqFfea0dXdd9vqai=hGuQ8kuc9pgc9s8qqaq=dirpe0xb9q8qiLsFr0=vr0=vr0dc8meaabaqaciaacaGaaeqabaqabeGadaaakeaacqWGWbaCcqGGOaakcqWGJbWycqGG8baFcqWGHbqycqGGPaqkcqGH8aapcqWGWbaCcqGGOaakcqWGJbWycqGGPaqkcqGHshI3daWcaaqaaiabdchaWjabcIcaOiabdogaJjabcYha8jabdggaHjabcMcaPaqaaiabdchaWjabcIcaOiabdogaJjabcMcaPaaacqGH8aapcqaIXaqmaaa@494F@

where p(c) is the probability that a region is conserved and p(c|a) is the probability that a region is conserved, given that it is an alpha helix.

We observed that the probability for a region to be an alpha helix, given that the region is conserved, is lower than the probability for a region to be an alpha helix in both conserved and random regions. This is formally defined by:

p(a|c)<p(a)⇒p(a|c)p(a)<1
 MathType@MTEF@5@5@+=feaafiart1ev1aaatCvAUfKttLearuWrP9MDH5MBPbIqV92AaeXatLxBI9gBaebbnrfifHhDYfgasaacH8akY=wiFfYdH8Gipec8Eeeu0xXdbba9frFj0=OqFfea0dXdd9vqai=hGuQ8kuc9pgc9s8qqaq=dirpe0xb9q8qiLsFr0=vr0=vr0dc8meaabaqaciaacaGaaeqabaqabeGadaaakeaacqWGWbaCcqGGOaakcqWGHbqycqGG8baFcqWGJbWycqGGPaqkcqGH8aapcqWGWbaCcqGGOaakcqWGHbqycqGGPaqkcqGHshI3daWcaaqaaiabdchaWjabcIcaOiabdggaHjabcYha8jabdogaJjabcMcaPaqaaiabdchaWjabcIcaOiabdggaHjabcMcaPaaacqGH8aapcqaIXaqmaaa@4947@

where p(a) is the probability of a region to be an alpha helix.

These two equations are consistent with Bayes' Theorem of conditional probability:

p(a|c)p(a)=p(c|a)p(c)
 MathType@MTEF@5@5@+=feaafiart1ev1aaatCvAUfKttLearuWrP9MDH5MBPbIqV92AaeXatLxBI9gBaebbnrfifHhDYfgasaacH8akY=wiFfYdH8Gipec8Eeeu0xXdbba9frFj0=OqFfea0dXdd9vqai=hGuQ8kuc9pgc9s8qqaq=dirpe0xb9q8qiLsFr0=vr0=vr0dc8meaabaqaciaacaGaaeqabaqabeGadaaakeaadaWcaaqaaiabdchaWjabcIcaOiabdggaHjabcYha8jabdogaJjabcMcaPaqaaiabdchaWjabcIcaOiabdggaHjabcMcaPaaacqGH9aqpdaWcaaqaaiabdchaWjabcIcaOiabdogaJjabcYha8jabdggaHjabcMcaPaqaaiabdchaWjabcIcaOiabdogaJjabcMcaPaaaaaa@450C@

Our findings that helices are less common in conserved regions than in random regions can therefore be explained by the fact that alpha helices can keep their tertiary structure without being highly conserved.

The same reasoning applies to our findings in beta strands. We find beta strands more likely to be conserved, and conserved regions are more likely to be beta strands given the overall occurrence of beta strands. This could be explained by the fact that strands tend to be conserved in order to keep their tertiary structure. It is generally accepted that beta-sheet formation, unlike that of alpha-helix, is to a large extent determined by tertiary context and not by the intrinsic properties of the residues in the strand [35]. Studies confirming this notion [18-20] have not shown that beta strands are generally more resistant to mutations than alpha helices. Therefore, the tertiary structure shown to be important for beta sheet stabilization may well be highly dependent on sequence.

It has been previously suggested by Bornberg-Bauer and Chan that proteins that have a large sequence neutral network – i.e. a large variety of sequences that can keep the initial structure – also tend to have a smooth folding funnel [36,23]. A smooth folding funnel ensures correct folding, without local minima that may lead to non-native conformations. By using simple exact lattice models, Bornberg-Bauer and Chan have shown that the size of the available sequence space correlates with folding energetics. In our study, we have shown a connection between actual sequence conservation and the number of available conformations (equivalent to density of states in the folding energy descriptions) as derived from secondary structure conformation constraints.

Protein folding paths are usually studied in light of structural constraints on single proteins. The native folding path is the one with "minimal frustration". Conservation emphasizes that proteins are the result of selective evolution. Mutations in a protein are usually fixed only if the protein function is not disturbed. Since a protein that cannot fold properly will not function, the propensity to fold correctly is an evolutionary constraint. If there are structural regions that can tolerate mutations, this increases the robustness of the protein in terms of structural stability and of function. Bornberg-Bauer and Chan suggested the term "superfunnels" for the sequence space that allows protein folding [23]. We suggest that the evolutionary selection for a specific secondary structure is affected by a reduction in structural degrees of freedom, which is inversely correlated with the "sequence degrees of freedom". This is apparent in our analysis of structure features in conserved regions, and by modeling the available protein space as shown by Bornberg-Bauer and Chan.

In other words, when a protein folds, there is an energetic price for losing entropy, namely, a loss of degrees of freedom. We claim that sequence constraints are a kind of entropic loss. Since strands lose a smaller number degrees of freedom just by being strands, they can afford to lose more degrees of freedom by sequence changes.

### Loops and turns – connectors, functional sites and structural stabilization

Loops were found to be common to a similar extent in conserved and random regions. This could be explained by the occurrence of loops in different contexts. Some loops are important for structure or function, and are therefore highly conserved within families as well as within superfamilies as previously shown by Liu et al. [10]. The conservation of loops should be especially high due to their inherent flexibility, which is higher than that of strands. Strand flexibility explains strand conservation as described above. Other loops might merely be connectors, so that their exact conformation is not important for protein function or structural stabilization. Therefore they are not conserved. If this is so, the occurrences of these two types of loops may cancel each other out when a large set of data is analyzed.

Turns are even more heterogeneous in context than loops, appearing in various types of structural positions such as the middle of loops, or adjacent to helices and strands. This diversity makes it difficult to identify the reasons for turns being less common in conserved regions.

## Conclusion

Sequence conservation indicates the structure and function of the whole protein. Secondary structure is strongly affected by local interactions and residue composition. Nonetheless, we have shown an association between secondary structure and conservation. The significant but small preference for strands, and avoidance of helices and turns in conserved regions implies that secondary structure is part of the interplay between sequence conservation and structural stabilization. Protein secondary structure represents a set of structural constraints that affect amino acid sequence constraints. This provides a better understanding of principles of protein folding, and evolutionary conservation of structure. Secondary structure constraints are also important determinants for combinations of SSEs in conserved sequence regions. These combinations can be identified as protein structure motifs that have specific functions [ES and SP, submitted].

## Methods

### Data collection and integration

The Blocks+ Database, versions 13.0 and 14.0, were used as a definition of conserved regions. The Blocks+ database is a database consisting of short ungapped sequence alignments automatically built using predefined protein families [2,27]. Protein families are predefined by the InterPro database [37], which consists of information from several sources. The block finding method used by Blocks is conservative, in order to avoid wrong block definitions, at the cost of possible loss of some correct blocks [4]. For each protein family a set of blocks is determined automatically, blocks range in length from 5 to 55 amino acids and include from 5 to 3100 sequences (most blocks are between 10 and 40 residues in length, and include between 5 and 90 sequences).

Kim Hendrick's SwissProt to PDB mapping, from EBI, was used to assign each conserved region's location on the structure as it appears in the PDB, and to identify resolved region [38]. Class, fold, and super-families of the domains relevant to the block were added from the SCOP database version 1.67 [11].

Secondary structure definitions are not always identical for homologous sequence, as well as for different structure solutions of the same protein (E. Sitbon, unpublished results). In order to assign a single secondary structure sequence to each conserved region, we first used the secondary structure assigned to each protein in the conserved region by the SwissProt database [39]. SwissProt uses an algorithm that defines a single secondary structure assignment from the best available structures based on DSSP [3] (Dr. Isabelle Phan (SP team), personal communication), assigning either strand, helix or turn to regions in the sequence. Helices include, 4-helices (alpha), 3_10 _helices and 5 helices (π). Strands include isolated beta bridges and extended strands, and turns include hydrogen bonded turns (Hbond(i,i+n), n = 3,4,5). Structurally resolved regions that do not fit into one of these categories were defined as loops. SSE lengths were ignored, that is, one element was assigned to a stretch of residues with an identical secondary structure definition.

**Figure 8:**
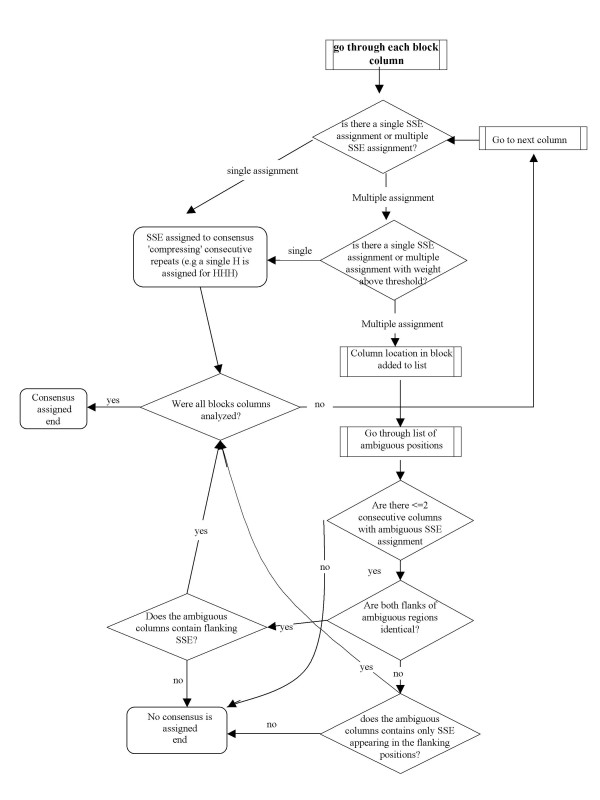
**Algorithm for creating an SSE consensus for a block**. The input is the SSE assignment for each protein region in the block. Rectangles represent a process, a diamond represents a decision. The algorithm ends either by assigning no consensus (NULL), or by assigning a specific consensus.

Where several proteins with defined structures were available for a block, each was given a position-based weight [40], and a SSE consensus was created by the algorithm described in Figure 8. When no consensus could be defined, the block was removed from the analysis, since losing some information was preferred to noise in the analysis. A few examples of generating SSE consensuses are shown in Table 5. A database was created integrating blocks accessions and sequences with PDB IDs, including chain and motif position on the PDB sequence, secondary structure assignment and SCOP information.

### Accessibility assignment

Accessibility, like secondary structures, varies between different crystallization experiments and between homologous sequences. The following steps were used to define an average accessibility value for each conserved region. For each region in the database, an average accessibility value was obtained by Naccess [41]. Naccess assigns a relative accessibility value to each amino acid. If this accessibility was above a threshold of 5%, the residue was considered accessible, the analysis was repeated with a threshold of 15% with similar results (data not shown). The average accessibility of the region was defined as the number of its accessible residues divided by the total number of residues in the region. Each protein may have more than one determined structure. The range of accessibilities from different crystal structures for each region of each protein was calculated (max accessibility – min accessibility). If the range was smaller than a threshold of 0.05, the region in that protein was assigned the average of all the accessibility values of the structures of the region of this protein. If the range was higher than the cutoff, no specific accessibility value was assigned for the region of the protein. In the next step, only conserved regions where all the available proteins with structure have an assigned accessibility value were examined. If the range of the different protein accessibility values was below the threshold of 0.01 the average of the average protein accessibilities was assigned to the conserved region. Similarly to SSE assignment, losing conserved regions information was preferred to excess noise when a large variation in accessibility was found between structures or proteins.

When analyzing each residue separately, a crystal structure (with the best resolution if several were available) was used for each block. Each residue was defined as accessible if its relative accessibility (from Naccess [41]) was over 5%. The secondary structure was defined by the single structure used, not by a consensus as in all other analyses. Unlike the analyses described above, here we counted the number of accessible residues in each SSE. Therefore, the number of residues was normalized by the total number of accessible residues.

### Creating background sets

Background sets of regions for SSE and accessibility calculations were generated from random protein regions. For each block, a random region was selected from the representative structure. Random regions were defined as regions with the same lengths as the conserved regions in the analysis. They were chosen in random locations on the protein sequence. The secondary structures of the randomly chosen segments were characterized in the same way as for the set of conserved regions (see above). The only difference was that the secondary structure was taken from one protein, without creating a SSE consensus. This random sampling and analysis was repeated twenty-two times (a large enough number arbitrarily chosen), which created a distribution of each SSE over all the sampled sets. For each subset of the blocks, such as a specific structural class, a corresponding random regions set was created.

### Statistical analysis

Several statistical tests were conducted to examine the significance of the analysis results. Distributions of secondary structure elements in conserved regions were compared to distributions in random regions using Chi square test for independence. This was done in order to test if the distribution between the four categories of secondary structures depends on whether or not the fragments are conserved.

In addition, a prediction interval was created in order to test the following null hypothesis: The number of each SSE type (helix, strand, turn or loop) in conserved regions belongs to the distribution of this SSE type in the sets of random regions with a 5% level of significance. The prediction interval was created using the t-value for alpha = 0.125(0.05/4) and correcting for multiple testing, and 21 degrees of freedom (corresponding to the twenty two samples minus one), multiplied by the standard deviation.

## Authors' contributions

ES collected the data, built the database, and created the analysis procedures. SP conceived of the study. Both authors participated in the design of the study and writing of the manuscript.

## Figures and Tables

**Table 1:** Statistics of sequence alignments used in this study

***Number of protein families***	***1401***
Number of blocks analyzed	4733
Number of analyzed SSE in blocks	30549
Number of amino acids in all blocks	118025
Average (median) block length	24.9(21)
Number of structurally resolved amino acids in sequences that contain blocks.	582402
Average (median) no. of resolved amino acids in sequences that contain blocks.	275.8(247)
Coverage of average single block length to resolved residues in a sequence	9%
Coverage of all blocks in resolved residues in all sequences	21%

**Table 2:** Secondary structure elements in conserved and random regions

**Elements**	**Number in blocks conserved regions (Observed)**	**Average number in random regions (Expected)**	**Cell X**^2 ^*
Helix	5537	5956	22.5
Strand	6846	6362	46.6
Turn	6252	6671	19.3
Loop	11914	11843	2.7

*Cell Chi square result. The total Chi square X^2^_(3)_= 91.2

**Table 3:** Statistics of secondary structure elements in conserved and random regions

**Elements**	**Difference (random average minus conserved)**	**% difference from conserved**	Number of prediction intervals *
**Helix**	**419.0**	**7.6**	**3.17**
**Strand**	**-483.6**	**-7.1**	**3.51**
**Turn**	**418.7**	**6.7**	**2.92**
Loop	-70.5	-0.6	0.36

* The number of prediction intervals that differentiates between conserved regions and the average of random regions. In bold lines the difference is significant with a confidence of at least 95%. Prediction interval was created by the standard deviation of the random sets multiplied by the t value for 21 degrees of freedom.

**Table 4:** Helix and strand occurrence in different structural classes

	**Conserved**	**Random**	Prediction interval *	**Difference**	Number of prediction intervals**
**Strands**					
Alpha/beta	2217	1984.4	109.2	-232.6	**2.13**
Alpha + beta	1556	1465.3	83.3	-90.7	**1.09**
All alpha	263	343.7	63.3	80.7	**1.27**
All beta	1666	1427.7	70.1	-238.3	**3.40**
**Helices**					
Alpha/beta	2127	2262.4	84.5	135.4	**1.60**
Alpha + beta	1109	1229.2	62.7	120.2	**1.92**
All alpha	1139	1104.2	37.3	-34.8	0.93
All beta	326	466.43	47.4	140.4	**2.97**

* Prediction interval was created by the standard deviation of the random sets multiplied by the t value for 21 degrees of freedom.

** The number of prediction intervals that differentiates between conserved regions and the average of random regions. In lines highlighted by bold print the difference was significant with a confidence of at least 95%.

**Table 5:** Examples for dealing with SSE variability in a block. Ambiguous regions are highlighted in bold and italics:

**SSEs in some blocks**	**Given consensus**	**Explanation**
HLL***SS***LLLL HLL***LL***LLLL HLL***HS***LLLL	HL	Identical flanks, ambiguous region contains flank SSE.
		
HLL***LL***TLLL HLL***LL***TLLL HLL***TT***TLLL	HLTL	Different flanks, ambiguous region contains only flank SSEs.
		
HLL***LL***TLLL HLL***LS***TLLL HLL***TT***TLLL	No consensus	Different flanks, strand is not one of the flank SSEs.
		
HLL***SSS***LLLL HLL***LLL***LLLL HLL***HSS*L**LLL	No consensus	Three consecutive ambiguous positions.
		
HLLT***S***TLLLL HLLT***H***TLLLL HLLT***L***TLLLL	No consensus	Identical flanks, ambiguous region does not contain flank SSE.
